# Tissue‐Specific Trisomy 10 Mosaicism Confirmed via Skin Fibroblast Analysis

**DOI:** 10.1002/cga.70078

**Published:** 2026-09-08

**Authors:** Kantaro Kimura, Shino Shimada, Hideo Fukunaga, Koki Nikai, Hiromichi Shoji

**Affiliations:** ^1^ Department of Pediatrics Juntendo University Faculty of Medicine Tokyo Japan; ^2^ Medical Genomics Center National Institute of Global Health and Medicine, Japan Institute for Health Security Tokyo Japan; ^3^ Department of Pediatric General and Urogenital Surgery Juntendo University Faculty of Medicine Tokyo Japan

Trisomy 10 mosaicism is extremely rare, and complete trisomy 10 is considered lethal; survival is possible only in mosaic cases [1, 2]. Reported cases of mosaic trisomy 10 showed a highly variable phenotype, including growth restriction, craniofacial dysmorphism, congenital heart defects, gastrointestinal anomalies, limb malformations, and neurodevelopmental impairment [3]. Because chromosomal mosaicism may be unevenly distributed across tissues, a normal peripheral blood karyotype does not exclude the possibility of a clinically suspected mosaic chromosomal abnormality.

The patient was a 14‐month‐old Japanese female infant conceived through artificial insemination. The mother was gravida 2, para 0, with no relevant medical history or medication use. Fetal growth restriction was identified at 16 weeks of gestation, and a ventricular septal defect and absence of the third left finger were detected at 20 weeks. Amniocentesis performed at 21 weeks revealed trisomy 10 mosaicism at 26 weeks, with a karyotype of 47,XX,+10[23]/46,XX[27]. She was referred to our hospital at 28 weeks of gestation, where fetal echocardiography suggested double‐outlet right ventricle with pulmonary stenosis. The baby was delivered via emergency cesarean section at 39 weeks and 3 days of gestation due to nonreassuring fetal status. Birth weight was 1906 g [−3.3 standard deviation (SD)], length was 45.0 cm (−2.1 SD), and head circumference was 29.8 cm (−2.6 SD), consistent with the symmetrical small‐for‐gestational‐age status. Apgar scores were 8 and 9 at 1 and 5 min, respectively. Physical examination revealed characteristic facial features, including low‐set ears, hypertelorism, ptosis, a broad forehead, a high‐arched palate, a broad nasal bridge, and micrognathia (Figure 1A). A split hand was present on the left side (Figure 1B). Echocardiography confirmed a double‐outlet right ventricle with a subaortic ventricular septal defect, pulmonary stenosis, and a persistent left superior vena cava. During the neonatal period, the patient developed bilious vomiting and was diagnosed with intestinal malrotation, for which surgical correction was performed on Day 10. Brain magnetic resonance imaging revealed no significant abnormalities. Auditory brainstem response testing demonstrated bilateral sensorineural hearing loss, and bronchoscopy revealed mild tracheobronchomalacia. Following the initiation of tube feeding, weight gain gradually improved, and the patient was discharged home at 3 months of age. At 4 months of age, cardiac catheterization revealed a pulmonary‐to‐systemic blood flow ratio of 2.4, a mean pulmonary artery pressure of 29 mmHg, and a pressure gradient of 45 mmHg across the pulmonary valve, indicating mild pulmonary hypertension and moderate pulmonary valve stenosis. The patient was managed conservatively with oral diuretics and remains under close follow‐up. At 5 months of age, she developed adhesive ileus requiring emergency surgery. A 35‐cm segment of partially ischemic small intestine was resected, followed by end‐to‐end anastomosis without complications. At 1 year of age, she was able to babble and sit independently and could tolerate small amounts of solid food while continuing tube feeding.

**FIGURE 1:**
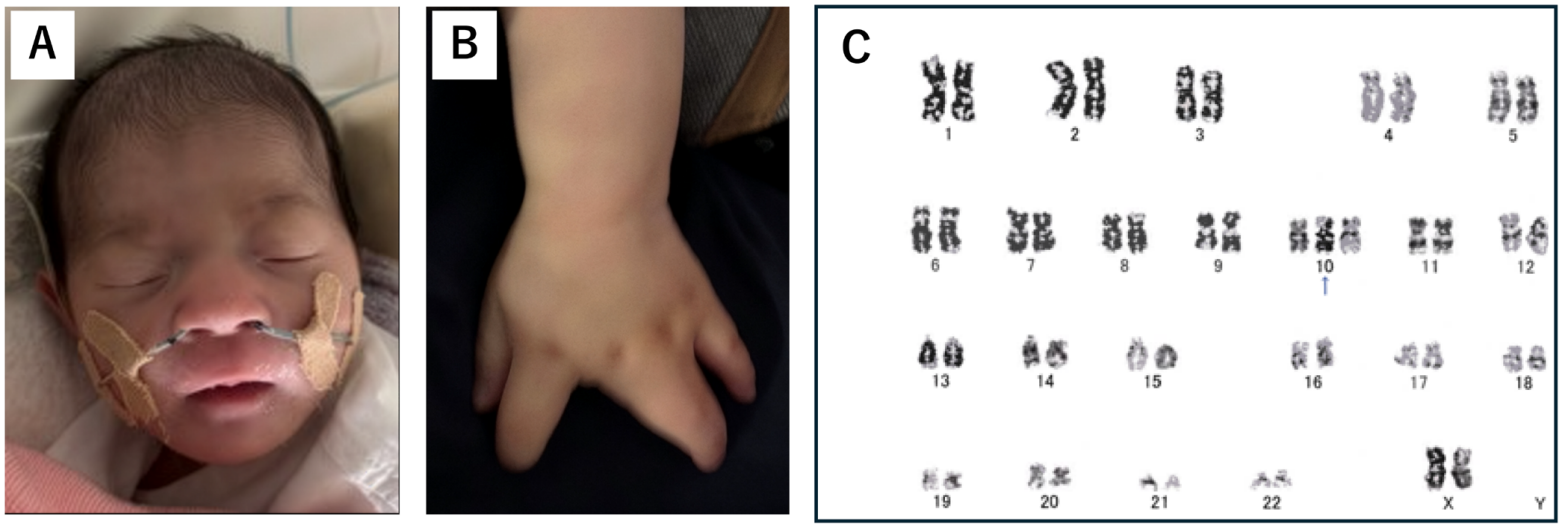
Clinical photographs of the patient. (A) Facial features, including low‐set ears, hypertelorism, a broad forehead, a broad nasal bridge, and micrognathia. (B) Split left hand. (C) G‐banding analysis of cultured skin fibroblasts. Karyotype analysis revealed 47,XX,+10 in 4 of 30 analyzed cells and 46,XX in the remaining 26 cells.

Postnatal cytogenetic testing was performed to confirm the prenatal diagnosis of trisomy 10 mosaicism. G‐banding analysis of peripheral blood lymphocytes on Day 4 revealed a normal female karyotype, 46,XX, in all 30 metaphases examined. Array comparative genomic hybridization of peripheral blood DNA detected no pathogenic copy number variants that could explain the phenotype. Fluorescence in situ hybridization of buccal mucosa cells using a chromosome 10 pericentromeric probe (D10Z1) demonstrated two signals in all 100 cells examined, providing no evidence of trisomy 10. Given the strong concordance between the prenatal karyotype and clinical phenotype, a skin biopsy was performed after informed parental consent was obtained. At 3 months of age, when the patient weighed 2800 g, a 5‐mm punch biopsy specimen was obtained from the buttock. G‐banding analysis of cultured skin fibroblasts revealed 47,XX,+10 in 4 of 30 cells and 46,XX in the remaining 26 cells, confirming trisomy 10 mosaicism with a mosaic ratio of 13% (Figure 1C). Genetic counseling indicated that mosaic trisomy 10 most likely resulted from a chromosomal nondisjunction event followed by cellular correction, such as a mitotic error or trisomy rescue.

Many such patients die during early infancy; among the eight reported survivors, two were diagnosed prenatally and six postnatally (Table S1) [3, 4, 5]. At 1 year, she could sit independently, indicating ongoing neurodevelopmental progress. In addition to the reported phenotypes, bilateral sensorineural hearing loss and tracheobronchomalacia were observed, representing additional manifestations. In this case, trisomy 10 mosaicism was identified prenatally in amniotic fluid, whereas postnatal analyses of peripheral blood lymphocytes and buccal mucosa were normal. The diagnosis was ultimately established after chromosome analysis of skin fibroblasts. Although useful for detecting low‐level mosaicism, this invasive test may not be the ideal first‐line detection method as detection varies by tissue, cell number, and method [6, 7]. Survival determinants remain unclear. One previously reported survivor without cardiac or gastrointestinal anomalies suggests that tissue‐specific mosaicism and organ involvement influence outcome. Accordingly, normal findings in a single tissue should be interpreted with caution when the clinical phenotype strongly suggests a mosaic chromosomal abnormality. Further accumulation of tissue‐specific cytogenetic data is needed to clarify the relationship between the level of mosaicism and clinical severity in trisomy 10 mosaicism. When prenatal testing indicates mosaic aneuploidy and the postnatal phenotype is consistent, normal findings in peripheral blood or buccal mucosa are insufficient to exclude mosaicism.

## Funding

The authors have nothing to report.

## Disclosure

The authors have read the CARE Checklist (2016), and the manuscript was prepared and revised in accordance with its recommendations.

## Ethics Statement

The authors have nothing to report.

## Consent

Written informed consent for the publication of this report and accompanying images was obtained from the patient's parents.

## Conflicts of Interest

The authors declare no conflicts of interest.

## Supporting information


**Table S1:** Summary of eight liveborn cases of trisomy 10 mosaicism.

## Data Availability

The data supporting the findings of this case report are available from the corresponding author upon reasonable request and subject to appropriate ethical approval.

## References

[1] M. L. Brizot , R. Schultz , L. T. Patroni , L. M. Lopes , E. Armbruster‐Moraes , and M. Zugaib , “Trisomy 10: Ultrasound Features and Natural History After First Trimester Diagnosis,” Prenatal Diagnosis 21, no. 8 (2001): 672–675, 10.1002/pd.129. 11536269

[2] J. Lévy , J. M. Jouannic , J. Saada , F. Dhombres , J. P. Siffroi , and M. F. Portnoï , “Prenatal Diagnosis of Bilateral Ectrodactyly and Radial Agenesis Associated With Trisomy 10 Mosaicism,” Case Reports in Genetics 2013 (2013): e592702, 10.1155/2013/592702. PMC3557612 23401811

[3] J. M. D. Hahnemann , M. N. M. Friberg , U. Engel , and M. Bugge , “Trisomy 10 Mosaicism and Maternal Uniparental Disomy 10 in a Liveborn Infant With Severe Congenital Malformations,” American Journal of Medical Genetics ‐ Seminars in Medical Genetics, Part A 138A, no. 2 (2005): 150–154, 10.1002/ajmg.a.30908. 16114048

[4] M. Higurashi , M. Naganuma , I. Matsui , and S. Kamoshita , “Two Cases of Trisomy C6‐12 Mosaicism With Multiple Congenital Malformations,” Journal of Medical Genetics 6, no. 4 (1969): 429–434, 10.1136/jmg.6.4.429. 5391902 PMC1468794

[5] Y. Gao , Y. C. Ma , Y. H. Ju , and Y. N. Li , “Mosaicism Trisomy 10 in a 14‐Month‐Old Child With Additional Neurological Abnormalities: Case Report and Literature Review,” BMC Pediatrics 18, no. 1 (2018): 266, 10.1186/s12887-018-1237-1. 30081864 PMC6090700

[6] M. Gajecka , “Unrevealed Mosaicism in the Next‐Generation Sequencing Era,” Molecular Genetics and Genomics 291, no. 2 (2016): 513–530, 10.1007/s00438-015-1130-7. 26481646 PMC4819561

[7] M. Silva , N. de Leeuw , K. Mann , et al., “European Guidelines for Constitutional Cytogenomic Analysis,” European Journal of Human Genetics 27, no. 1 (2019): 1–16, 10.1038/s41431-018-0244-x. 30275486 PMC6303289

