# Supplementary material for: Tissue‐Specific Trisomy 10 Mosaicism Confirmed via Skin Fibroblast Analysis
Source: Congenit Anom (Kyoto). 2026 Sep 8;66(1):e70078. doi: 10.1002/cga.70078 (PMC13553155; doi:10.1002/cga.70078)
Supplement: Supplementary file 1 [file CGA-66-0-s001.docx]

| **Supplemental Table 1. Summary of eight liveborn cases of trisomy 10 mosaicism.** | | | | | | | | |
| --- | --- | --- | --- | --- | --- | --- | --- | --- |
| **Author (year)** | **Higurashi**  **(1969)^4^** | **Qazi**  **(1976)** | **Vianello**  **(1978)** | **De France**  **(1985)** | **Mielke**  **(1997)** | **Hahnemann**  **(2005)^3^** | **Gao**  **(2018)^5^** | **Present case** |
| Maternal age (years) | 31 | Mid-20s | 19 | 33 | 26 | 32 | 34 | 34 |
| Sex | M | F | M | M | F | M | F | F |
| Birth weight (g)/gestational age (weeks) | 3,150/40 | 2,300/36 | 3,000/40 | 1,930/41 | 920/36 | 1,760/37 | 1,900/41 | 1,906/39 |
| Prognosis | Alive (5 years and 4 months) | NR | Died at 6 months | Died at 7 days | Died at 7 weeks | Died at 37 days | Alive (1 year and 5 months) | Alive (1 year) |
| Mosaic ratio in peripheral blood (%) | 39 | 33 | 28 | 0 | 0 | 6 | 42 | 0 |
| Mosaic ratio in skin fibroblasts (%) | 55 | 33 | 20 | 83 | 94 | 83 | NR | 13 |
| Growth restriction | + | + | + | + | + | + | + | + |
| Craniofacial dysmorphism | + | + | + | + | + | + | + | + |
| Cardiac findings | − | + | + | Complete AV block | Single atrium | ASD, VSD | ASD, PDA | DORV |
| Gastrointestinal malformations | − | − | − | − | − | Intestinal malrotation | − | Intestinal malrotation |
| Limb anomalies | + | + | + | – | + | + | + | + |
| Neurodevelopmental impairment | + | NR | + | NR | NR | NR | + | + |
| Abbreviations: ASD, atrial septal defect; AV, atrioventricular; DORV, double-outlet right ventricle; NR, not reported; PDA, patent ductus arteriosus; VSD, ventricular septal defect. | | | | | | | | |
